# Poorly Regulated Antibiotic Use in Nigeria: A Critical Public Health Concern and Its Impact on Medical Practice

**DOI:** 10.7759/cureus.85212

**Published:** 2025-06-01

**Authors:** Olajide J Olagunju, Egbo Ben, Olayinka Olagunju, Oluwadamilola G Majolagbe, Olagoke O Osanyinlusi, Titilade Adewoye, Omolola F Atoyebi, Iyanuloluwa O Ojo, Sam D Dawha

**Affiliations:** 1 Division of Infectious Disease, Case Western Reserve University School of Medicine, Cleveland, USA; 2 Department of Biostatistics and Epidemiology, East Tennessee State University, Johnson City, USA; 3 Department of Radiology, Potiskum Medical Center, Potiskum, NGA; 4 Department of Zoology, Obafemi Awolowo University, Ile-Ife, NGA; 5 Department of Nursing, University of Ottawa, Overland Park, USA; 6 Department of Anesthesiology, Washington University School of Medicine in St. Louis, St. Louis, USA; 7 Department of Sustainability and Environmental Management, Coventry University, Coventry, GBR; 8 Department of Nursing, Barnes Jewish Hospital, St. Louis, USA; 9 Department of Dentistry, Maryborough Hospital, Queensland Health, Maryborough, AUS; 10 Department of Radiology, Federal University of Health Sciences Teaching Hospital, Azare, NGA

**Keywords:** antibiotic misuse, antimicrobial resistance, nigeria, pharmacovigilance, self-medication

## Abstract

Antibiotics have transformed the treatment of infectious diseases, but their widespread misuse has fueled an alarming rise in antimicrobial resistance (AMR), especially in low- and middle-income countries like Nigeria. Despite regulatory frameworks classifying antibiotics as prescription-only medicines, weak enforcement, self-medication, and informal drug sales have normalized inappropriate antibiotic use nationwide.

A comprehensive narrative review was conducted, synthesizing evidence from peer-reviewed literature, surveillance reports from national and global health authorities, and policy documents. The review identifies key systemic, socioeconomic, and behavioral drivers of antibiotic misuse and their implications for clinical practice.

Multiple factors, including weak pharmaceutical regulation, economic and geographic barriers to formal healthcare, public misconceptions, and poor pharmacovigilance, drive the misuse of antibiotics in Nigeria. These practices contribute to rising rates of multidrug-resistant infections, complicate routine procedures, increase treatment costs, and lead to diagnostic uncertainty. Clinicians face growing professional burnout due to repeated treatment failures and limited therapeutic options. The broader healthcare system is strained, and public trust in formal care is eroding.

AMR in Nigeria is no longer a looming threat, it is a present crisis. Urgent, coordinated action is required to strengthen regulatory enforcement, improve healthcare access, implement antimicrobial stewardship, and invest in public health education. Without immediate intervention, Nigeria risks entering a post-antibiotic era where routine infections become life-threatening and modern medicine becomes increasingly ineffective.

## Introduction and background

Scientists discovered antibiotics in the 20th century, revolutionizing the treatment of infectious diseases and saving countless lives. However, the widespread misuse and overuse of these powerful drugs have now fueled a growing global crisis of antimicrobial resistance (AMR) [1]. This threat looms especially large in low- and middle-income countries like Nigeria, where weak regulatory systems, indiscriminate use, and poorly monitored sales of antibiotics have triggered a serious public health emergency [2]. These practices now endanger individual patients and the broader population, with consequences extending far beyond national borders.

In Nigeria, people frequently obtain antibiotics without prescriptions, sourcing them from informal vendors, community pharmacies, or over-the-counter outlets with minimal regulatory oversight [3]. Many individuals self-medicate, use incorrect dosages, stop treatment prematurely, or take antibiotics for viral or undiagnosed conditions. Poverty, poor access to qualified healthcare providers, and widespread lack of awareness continue to drive these practices. Although they may appear necessary in resource-limited settings, these behaviors collectively cause significant harm. They promote the emergence and spread of resistant pathogens, weaken the effectiveness of standard treatments, and place additional strain on an already overstretched healthcare system [2].

Medical practitioners across Nigeria now face serious consequences from rising antibiotic resistance. Clinicians increasingly encounter multidrug-resistant infections that prove difficult and at times impossible to treat with first-line antibiotics [4,5]. Routine procedures such as cesarean section, appendectomy, wound care, and the management of common infections like pneumonia or urinary tract infections (UTIs) have become more complicated and expensive due to frequent treatment failures [6,7]. This decline in antibiotic effectiveness threatens to reverse decades of medical progress and forces Nigerian healthcare providers to navigate a difficult landscape, meeting patient expectations while contending with limited diagnostic tools, shrinking treatment options, and widespread systemic challenges.

This narrative review critically examines the poorly regulated use of antibiotics in Nigeria, identifying its root causes, assessing its impact on clinical practice and public health, and evaluating the escalating threat of drug-resistant infections. It highlights this often-overlooked crisis to underscore the urgent need for coordinated interventions, stricter policy enforcement, and widespread public education. By addressing these challenges, Nigeria can preserve the effectiveness of antibiotics and safeguard the future of medical care nationwide.

Aim

(1) To critically examine the root causes, public health consequences, and clinical impact of poorly regulated antibiotic use in Nigeria.

(2) To explore how these practices contribute to the emergence of drug-resistant infections and undermine medical care across the country.

(3) To highlight the urgent need for coordinated interventions to curb AMR and preserve the effectiveness of medical treatments.

## Review

The Nigerian reality: Antibiotics without boundaries

Across Nigeria, people can easily buy antibiotics from virtually any corner, open markets, roadside stalls, or small neighborhood drug shops without ever seeing a doctor or pharmacist [3]. Although Nigerian law officially classifies antibiotics as prescription-only medicines, enforcement remains weak, and the reality is starkly different. Medications like amoxicillin, ciprofloxacin, metronidazole, tetracycline, and every other antibiotic circulate freely and are sold without prescriptions, proper dosing instructions, or professional guidance. In both urban centers and remote villages, street vendors, patent medicine dealers, and loosely supervised pharmacies serve as the primary sources of antibiotics for millions [3]. In numerous communities, vendors sell antibiotics in loose sachets or broken blister packs, without labeling, dosage instructions, expiration dates, or proper storage conditions. These practices not only violate established pharmaceutical standards but also place patients at serious risk of adverse outcomes.

Even more alarming is the circulation of counterfeit or substandard antibiotics within the supply chain, especially in informal markets. These poor-quality medications may contain incorrect active ingredients, insufficient drug concentrations, or contaminants, leading to ineffective treatment, prolonged illness, and the accelerated development of AMR. These informal providers often operate beyond the oversight of national regulatory bodies, leaving a gaping hole in the country’s pharmaceutical control system. As a result, the unchecked availability of antibiotics fuels widespread misuse, with dangerous implications for both individual health and national efforts to combat AMR.

**Figure 1:**
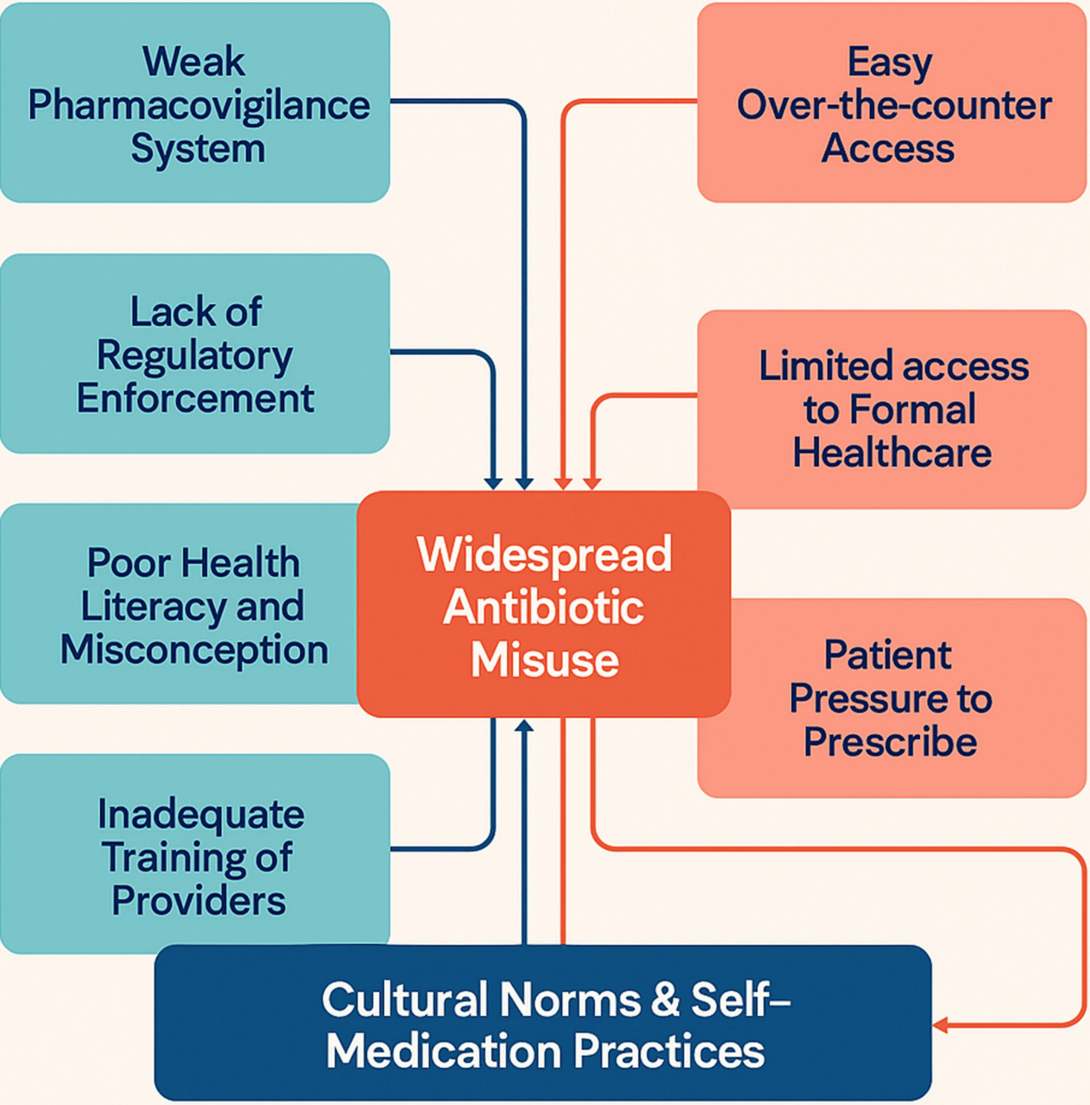
Complex Interplay of Factors Contributing to Antibiotic Misuse in Nigeria Image Credit: Created by Olajide J. Olagunju

Multiple factors drive the widespread misuse of antibiotics in Nigeria, as described in Figure 1, but at the core lies poor accessibility and limited affordability of formal healthcare. Many Nigerians, especially in low-income or rural communities, struggle to access hospitals or afford a visit to a licensed medical provider. Government hospitals are often overcrowded, with long queues that demand hours of waiting, while private clinics charge consultation fees and diagnostic costs that are far beyond what many families can afford [8]. Faced with these barriers, people turn to what feels like their only options, self-diagnosis and self-medication. Relying on personal experience, symptom matching, or advice from friends, relatives, or informal drug sellers, they often buy antibiotics directly over the counter. This behavior has become so common that many view it as a practical and cost-saving alternative to professional care. However, this “trial and error” approach puts patients at risk and fuels a dangerous cycle of incomplete treatments, misused medications, and growing resistance.

Widespread misinformation and limited public understanding continue to fuel the misuse of antibiotics across Nigeria [9,10]. Many people in both urban and rural communities believe that antibiotics can cure virtually any illness, from malaria and diarrhea to the common cold and body pain, regardless of whether the condition is bacterial, viral, or self-limiting. This misconception leads individuals to purchase and take antibiotics without knowing whether the drug is appropriate, effective, or even necessary [3,9,10]. They often stop taking the medication once symptoms improve. This practice exposes pathogens to subtherapeutic doses, allowing the strongest ones to survive and develop resistance. In effect, each incomplete or inappropriate use of antibiotics becomes an opportunity for dangerous bacteria to evolve, adapt, and ultimately render these life-saving drugs ineffective for future infections. Without urgent public education and awareness campaigns, this cycle of misuse will continue to undermine both individual treatment outcomes and national efforts to control AMR.

Together, these unchecked practices have created an environment in Nigeria where people use antibiotics freely, frequently, and often dangerously without any professional guidance or regulation. Vendors dispense medications casually, patients self-medicate without understanding the consequences, and many healthcare outlets operate outside formal oversight. This widespread misuse leads to more than just poor health outcomes; it delivers ineffective treatment to millions of Nigerians and fuels a silent but accelerating epidemic of AMR. Day by day, bacteria are evolving to withstand the very drugs meant to kill them, rendering once-reliable antibiotics powerless. If stakeholders fail to act swiftly through coordinated reforms involving public health authorities, regulatory agencies, healthcare providers, and communities, the consequences could be catastrophic. Nigeria risks entering a healthcare era where common infections no longer respond to standard treatments, and simple medical procedures become high-risk interventions.

Root causes of poorly regulated antibiotic use in Nigeria

Rampant antibiotic misuse in Nigeria and the accelerating threat of AMR result from a deeply rooted convergence of systemic breakdowns and sociocultural practices. Addressing this public health crisis demands more than isolated interventions; it requires a clear and comprehensive understanding of the underlying forces that fuel unregulated antibiotic consumption. These drivers include weak regulatory oversight, widespread misinformation, limited access to quality healthcare, and ingrained behaviors that normalize self-medication. Tackling these root causes is crucial to reversing the tide of resistance and protecting the future of effective medical treatment in Nigeria.

Inadequate Regulation/Enforcement

Although Nigerian law classifies antibiotics as prescription-only medications, enforcement remains largely ineffective in practice. Regulatory bodies like the National Agency for Food and Drug Administration and Control (NAFDAC) and the Pharmacists Council of Nigeria (PCN) face significant challenges, including limited funding, inadequate staffing, and logistical hurdles that severely undermine their reach and impact [11,12]. As a result, thousands of drug outlets, including informal vendors, street kiosks, and patent medicine stores, dispense antibiotics without prescriptions, proper oversight, or adherence to established safety standards. Many of these outlets operate without licensed pharmacists or medically trained personnel, creating a regulatory void that enables unchecked sales and unsafe dispensing practices. This persistent failure to enforce pharmaceutical regulations has rendered antibiotics dangerously accessible and misused, accelerating the rise of drug-resistant infections and weakening the foundation of effective medical treatment. This systemic lapse not only reflects gaps in policy execution but also highlights the urgent need for regulatory reform.

Economic and Geographic Barriers

Widespread economic hardship and persistent geographic barriers prevent millions of Nigerians from accessing formal healthcare services [13,14]. In underserved rural and peri-urban communities, government hospitals often suffer from overcrowding, staff shortages, and limited diagnostic capacity, resulting in long wait times and delayed care [8]. Meanwhile, private clinics and diagnostic centers, though more efficient, remain financially out of reach for many low-income families. Faced with these structural obstacles, individuals frequently resort to self-medication not out of ignorance or disregard, but as a matter of survival and practicality. For most, seeking help from a nearby drug vendor or patent medicine store offers a quicker, more affordable path to symptom relief than navigating the formal healthcare system. These informal providers rarely require prescriptions, charge minimal fees, and offer treatment recommendations based on anecdotal experience rather than clinical training. This pattern of reliance on unregulated drug outlets reflects a deeper issue: the exclusion of large segments of the population from accessible, affordable, and timely medical care. As a result, Nigeria’s informal pharmaceutical landscape has become a breeding ground for unregulated antibiotic use, where economic desperation intersects with systemic failure.

Widespread Misconceptions and Knowledge Gaps Among Patients and Providers

A deep-seated lack of public understanding about antibiotics continues to fuel widespread misuse across Nigeria. Many individuals mistakenly view antibiotics as universal remedies for any illness, whether bacterial, viral, or undiagnosed [9,10,15]. In numerous communities, people routinely turn to antibiotics to treat malaria, the common cold, fever, diarrhea, and generalized body pain, conditions that often do not require antibiotic therapy [9]. These beliefs, shaped by anecdotal experiences, community norms, and a lack of public health education, normalize self-medication and promote inappropriate drug choices. As a result, people often take antibiotics in incorrect doses, combine multiple antibiotics unnecessarily, or discontinue treatment as soon as symptoms subside, behaviors that promote subtherapeutic exposure and drive the emergence of resistant strains. The problem is not limited to the public alone. Knowledge gaps also persist among healthcare providers, particularly in informal or low-resource settings. Some clinicians prescribe antibiotics without diagnostic confirmation or clear clinical indication, relying instead on habit, patient pressure, or outdated medical training. In some cases, providers operate without access to continuing medical education, updated prescribing guidelines, or diagnostic tools that support rational antibiotic use. These gaps in professional practice, whether due to training deficiencies, systemic overload, or insufficient supervision, reinforce the culture of misuse and amplify the spread of AMR.

Clinical Pressures and the Culture of Defensive Prescribing

Clinicians across Nigeria often navigate a challenging clinical environment shaped by limited resources and high patient expectations. In both public and private settings, healthcare providers face constant pressure from patients and their families, who expect immediate relief and tangible treatment, often in the form of medication. With diagnostic tools and laboratory services frequently unavailable, delayed, or unaffordable, many clinicians resort to empirical treatment strategies. In these scenarios, antibiotics become a default choice not because they are always clinically appropriate, but because they offer a perceived safeguard against worsening illness or perceived inaction. Physicians, particularly in overcrowded clinics or rural hospitals, may feel compelled to prescribe antibiotics "just in case" to avoid clinical deterioration, meet patient expectations, or protect themselves from complaints or legal repercussions. This pattern reflects a widespread culture of defensive medicine, where antibiotics serve as a fallback solution in the face of diagnostic uncertainty. While often well-intentioned, these prescribing habits quietly fuel the misuse of antibiotics and the escalation of AMR. Over time, the normalization of antibiotics as a catch-all solution erodes clinical rigor and undermines public trust in evidence-based care.

Weak Pharmacovigilance and Surveillance Systems

As indicated in Figure 1, Nigeria’s capacity to monitor and respond to antibiotic misuse remains inadequate due to an underdeveloped pharmacovigilance system [16,17]. Health authorities and institutions struggle to track patterns of antibiotic consumption, detect resistance trends, or document adverse drug reactions in a timely and reliable manner. Most healthcare facilities lack dedicated personnel, reporting structures, or digital platforms to systematically collect and share data on antimicrobial use. As a result, information on antibiotic prescribing remains fragmented, inconsistently reported, and poorly centralized across regions and institutions. This data vacuum severely impairs the ability to develop and implement evidence-based interventions. Without routine antimicrobial stewardship programs or real-time surveillance systems, most hospitals operate without clear insights into resistance trends or emerging threats. Clinicians are left to make prescribing decisions without access to local antibiograms or resistance maps, and policymakers must navigate the crisis without a firm grasp of its scale or evolution. Without robust pharmacovigilance, Nigeria remains reactive rather than proactive in addressing AMR. This lack of actionable data limits opportunities for early intervention, resource prioritization, and accountability, allowing the silent spread of resistance to go unchecked.

The escalating threat of AMR 

AMR has emerged as one of the most urgent global health threats of the 21st century, undermining decades of medical progress [18-20]. As microorganisms increasingly develop resistance to the drugs designed to kill them, once-treatable infections are becoming harder, and sometimes impossible to cure. In simpler terms, AMR occurs when germs evolve in such a way that antibiotics and other antimicrobial agents become ineffective against them. This makes infections harder to treat, increases the risk of disease spread, severe illness, and death, and burdens health systems with higher costs and longer hospital stays. In countries like Nigeria, where antibiotics are frequently accessed without prescriptions and stewardship systems remain weak, the unchecked spread of resistant pathogens is fueling a silent public health crisis. AMR not only endangers individual patient outcomes but also threatens the effectiveness of modern medicine, increases healthcare costs, and poses significant risks to global health security. Urgent, coordinated efforts are needed to curb this escalating threat and safeguard the efficacy of life-saving treatments for future generations. 

Furthermore, it is important to understand that AMR is not a result of the body becoming resistant to medicine; rather, it is the microbes themselves that develop resistance [5]. This resistance can develop through multiple ways, including the misuse of antibiotics (e.g., taking them for viral infections like colds), not completing prescribed doses, and excessive or inappropriate use in agriculture and livestock. Resistant microorganisms can then spread within communities, hospitals, and across national borders, making AMR a growing global health challenge.

Nigeria is experiencing an alarming rise in AMR, as highlighted by multiple surveillance reports from the Nigeria Centre for Disease Control (NCDC) and the World Health Organization (WHO) [21]. According to the NCDC’s “Antimicrobial Use and Resistance in Nigeria” report, resistance is notably high in pathogens such as *Escherichia coli*, *Klebsiella pneumoniae*, *Staphylococcus aureus*, and *Pseudomonas aeruginosa* [22]. These bacteria have shown resistance to commonly used antibiotics, including third-generation cephalosporins, fluoroquinolones, and even carbapenems, which are considered last-resort antibiotics.

For example, surveillance data from 2022 revealed that over 70% of *E. coli* isolates were resistant to ciprofloxacin, and more than 60% of *K. pneumoniae* isolates showed resistance to ceftriaxone [23]. These trends are compounded by unregulated over-the-counter sales of antibiotics, widespread self-medication, and limited diagnostic infrastructure in many healthcare settings. Furthermore, healthcare professionals often rely on empirical treatment in the absence of laboratory guidance, leading to inappropriate prescribing and further promoting resistance.

The WHO's Global Antimicrobial Resistance Surveillance System (GLASS) has identified Nigeria as a priority country for AMR containment, citing weak regulatory systems, insufficient laboratory capacity, and inadequate data reporting as major challenges [24]. However, Nigeria has taken steps toward addressing AMR, including the development of a National Action Plan on AMR (2017-2022) and the implementation of antimicrobial stewardship programs in select tertiary hospitals.

Global Implications: Nigeria’s Role in the Global Health Network

Nigeria’s AMR crisis is not an isolated issue but part of a larger global health concern. The country’s large population, high disease burden, and significant levels of antimicrobial misuse make it a critical node in the global AMR network. Resistant organisms that emerge in Nigeria can spread internationally through travel, trade, and migration, contributing to the global transmission of superbugs. This means that localized resistance patterns in Nigeria can have far-reaching consequences, especially in our highly interconnected world.

Furthermore, Nigeria is considered a regional health hub in sub-Saharan Africa. Its ability or failure to manage AMR effectively could set a precedent for neighboring countries. The spread of resistant infections across borders can compromise the effectiveness of essential medical procedures worldwide, including surgeries, chemotherapy, and organ transplants, all of which depend on reliable antibiotics for infection prevention and control.

Globally, the rise in AMR threatens to reverse decades of medical progress and could lead to an estimated 10 million deaths annually by 2050 if no action is taken. The economic burden is also staggering, with projections estimating a loss of up to $100 trillion in global output [25]. In this context, Nigeria's active participation in global AMR containment efforts through surveillance, regulation, research, and public health education is essential. Collaboration with international partners, such as the WHO, CDC, and global health NGOs, will be key in building resilient systems capable of curbing this growing threat.

AMR is a complex and escalating threat that demands urgent action at both national and international levels. Nigeria, due to its strategic position and public health dynamics, holds a pivotal role in the global fight against AMR. Strengthening surveillance, enforcing regulations, promoting rational drug use, and investing in health system capacity are critical steps toward containing AMR and protecting future generations.

Consequences of poorly regulated antibiotic use on medical practice in Nigeria

Diagnostic Confusion Due to Partial Treatment

Since many individuals in Nigeria initiate antibiotic treatment without consulting a qualified healthcare provider, they often rely on leftover medications from past illnesses, outdated prescriptions, or advice from untrained sources such as drug vendors and neighbors. This self-medication typically involves inappropriate drug selection, incorrect dosages, or incomplete treatment courses, practices driven by economic hardship, convenience, and widespread misconceptions about how antibiotics work. By the time these patients finally present at a healthcare facility, they have already altered the natural course of their illness. Symptoms such as fever may have temporarily subsided, inflammation may appear reduced, and the characteristic signs of infection are often partially masked, complicating diagnosis and delaying appropriate medical intervention.

These changes present major diagnostic challenges for clinicians. Laboratory tests such as blood and urine cultures, which are critical for identifying the causative organisms, frequently yield false-negative results after prior antibiotic exposure [26,27]. The blunted clinical picture can mislead even experienced providers, who must now navigate a murky diagnostic landscape with limited reliable information. In many low-resource settings, where access to advanced diagnostic tools is already constrained, this uncertainty forces healthcare workers to make decisions based on guesswork rather than evidence-based assessments.

Consequently, treatment is delayed or misdirected, putting patients at risk of receiving ineffective or unnecessary therapies. In some cases, the delay in accurate diagnosis results in complications, prolonged illness, or the escalation of care to more intensive and expensive interventions. This not only jeopardizes patient outcomes but also frustrates healthcare professionals, who must grapple with the consequences of decisions shaped by incomplete or misleading clinical information. Ultimately, widespread misuse of antibiotics erodes the clinician’s ability to provide safe, timely, and effective care. It undermines the diagnostic process, distorts the presentation of infectious diseases, and contributes to a cycle of therapeutic failure and resistance. Addressing this challenge requires a multifaceted approach, one that includes public education, better access to diagnostics, and stricter regulation of antibiotic distribution to preserve the integrity of clinical decision-making.

Resistant Infections in Hospitals

Hospitals across Nigeria are facing a growing crisis as antibiotic-resistant infections become increasingly common among patients with typhoid fever, UTIs, respiratory tract infections, and sepsis [2,4,7,10]. Clinicians now confront pathogens that no longer respond to first-line antibiotics such as ampicillin, ciprofloxacin, or co-trimoxazole, drugs that were once considered dependable and affordable. Instead, they must turn to second- or third-line therapies like carbapenems and advanced-generation cephalosporins. These alternatives are not only significantly more expensive but also difficult to procure, particularly in under-resourced facilities where pharmaceutical supply chains are fragmented and erratic.

This shift in the treatment approach places a substantial burden on the healthcare system [28]. The need for more potent antibiotics leads to increased costs for patients, most of whom pay out-of-pocket, and prolongs hospital admissions, further straining already overcrowded wards. In many cases, the lack of access to appropriate drugs results in treatment delays or outright failure, raising the risk of complications, permanent disability, or death. For critically ill patients, especially in emergency settings or intensive care units, these delays can be fatal. The presence of multidrug-resistant (MDR) organisms also fuels the risk of hospital-acquired infections and localized outbreaks. Patients with compromised immune systems, such as the elderly, newborns, or those with chronic conditions, are particularly vulnerable. The infection control infrastructure in many Nigerian hospitals is inadequate to contain the spread of such organisms, leading to cross-infections, repeated admissions, and, in some cases, the closure of hospital units to prevent further transmission.

What was once a global public health warning has become a daily clinical reality in Nigeria. AMR is no longer an abstract concept discussed in policy circles or international forums; it now directly undermines the ability of healthcare providers to deliver safe, effective care. Each resistant infection represents not just a treatment failure but also a systemic failure, highlighting the urgent need for national action, stronger antibiotic stewardship, and investment in laboratory diagnostics and infection control.

Increased Cost of Care Due to MDR Organisms

Antibiotic resistance dramatically amplifies the financial burden on both individual patients and Nigeria’s already strained healthcare system, with health insurance covering only about 19% of the total population [29]. When standard first-line treatments fail due to resistance, clinicians have no choice but to prescribe more expensive second- or third-line antibiotics. These alternatives, such as carbapenems or advanced cephalosporins, are often not only harder to obtain but also far more costly, placing them out of reach for many low-income families. The economic impact extends beyond medication. Managing resistant infections demands additional diagnostic testing, prolonged hospital stays, and increased clinical supervision, all of which drive up healthcare costs significantly.

In Nigeria, where health insurance coverage is minimal and most patients pay for care out of pocket, these escalating costs quickly become unmanageable. Families who live on modest or unstable incomes frequently face heartbreaking choices: continue expensive medical treatment or redirect limited resources toward food, rent, or school fees. For many, the financial strain forces them to abandon treatment prematurely or avoid healthcare altogether, often turning instead to cheaper, informal options that further fuel inappropriate antibiotic use. These delays and disruptions in care not only compromise patient outcomes but also contribute to the spread of resistant infections across communities.

On a systemic level, the increased cost of care for resistant infections diverts limited hospital resources away from other essential services, weakening the broader healthcare infrastructure [30]. Beds remain occupied longer, laboratory and pharmacy demands increase, and overstretched health workers are burdened with more complex cases. This growing economic pressure underscores the urgency of implementing national antibiotic stewardship programs, subsidizing essential medicines, and expanding affordable healthcare coverage to protect vulnerable populations from the dual threat of disease and financial catastrophe.

Erosion of Public Trust When Treatment Fails

When antibiotic treatments fail, despite being properly prescribed by qualified healthcare professionals, many patients and their families respond with frustration, confusion, and an erosion of trust in the medical system. These reactions are not merely emotional; they are shaped by years of systemic inadequacies, poor health communication, and inconsistent care experiences. In a country where many communities already perceive formal healthcare as inaccessible, ineffective, or indifferent, treatment failure becomes a tipping point. Rather than recognizing the role of AMR or systemic factors beyond the clinician’s control, patients often attribute poor outcomes to medical incompetence, misdiagnosis, or low-quality drugs. Some may openly accuse healthcare providers of negligence or malpractice, while others quietly lose faith in professional care and turn back to informal and unregulated alternatives.

This erosion of trust has profound consequences. Patients who once sought care in hospitals may begin to avoid them entirely, relying instead on self-medication, local chemists, or traditional healers. These alternatives often lack scientific validation and may involve harmful or ineffective remedies, but they are perceived as more accessible, affordable, and emotionally reassuring. In some cases, families abandon treatment prematurely when symptoms fail to resolve quickly or when repeat hospital visits become financially and emotionally draining. Over time, this distrust entrenches a dangerous cycle: patients delay care, attempt home-based remedies, worsen, and finally present at health facilities with advanced disease, often after contributing to the spread of resistant pathogens in the community.

Clinicians, caught in this dynamic, face an increasingly difficult landscape. They may feel pressured to prescribe antibiotics even when unnecessary, simply to meet patient expectations or avoid confrontation. Others may default to broad-spectrum agents preemptively, fearing the backlash of another failed therapy. This culture of defensive medicine, driven not just by fear of repercussions but by the need to preserve professional credibility, further fuels antibiotic misuse and accelerates resistance. Moreover, it distracts attention from evidence-based practice and undermines the clinician's ability to educate patients on rational drug use.

Rebuilding trust in the healthcare system is not just a moral imperative; it is a strategic necessity in the fight against AMR. Public health interventions must move beyond awareness campaigns and begin fostering genuine patient-provider relationships grounded in empathy, accountability, and transparency. Healthcare workers must be equipped not only with clinical tools but also with communication skills to explain treatment failures, set realistic expectations, and involve patients in shared decision-making. Likewise, communities must be empowered with accurate information about antibiotics, resistance, and the importance of completing treatments and reporting adverse outcomes. Without restoring this trust, even the most robust antimicrobial policies and guidelines will struggle to succeed. In a context where misinformation thrives and care is often transactional, reclaiming trust could be the most potent tool in reversing the tide of antibiotic misuse and resistance.

Burden on Healthcare Providers Who Can’t Rely on First-Line Drugs

As antibiotic resistance grows, healthcare providers across Nigeria face mounting pressure and professional frustration due to the diminishing effectiveness of commonly prescribed antibiotics. When first-line drugs such as ampicillin, ciprofloxacin, or co-trimoxazole fail to treat infections effectively, clinicians are forced to consider more complex, expensive, and often inaccessible alternatives. This challenge becomes especially daunting in rural or under-resourced health facilities, where diagnostic laboratories are either rudimentary or nonexistent, and where access to second- or third-line antibiotics is limited or unaffordable. In such settings, providers must make urgent decisions without the benefit of microbial sensitivity testing, relying instead on clinical judgment and guesswork.

Emergency scenarios further magnify this burden. When faced with critically ill patients who may be septic or battling drug-resistant urinary or respiratory tract infections, clinicians find themselves racing against time. Yet, their therapeutic arsenal is increasingly ineffective. The uncertainty around which antibiotics will work, and whether any will at all, creates immense cognitive and emotional stress. Every treatment decision carries the risk of failure, complications, or even loss of life, placing an invisible but heavy toll on the provider.

This persistent uncertainty erodes the very foundation of clinical confidence. Over time, physicians, pharmacists, and nurses begin to question their own effectiveness, especially when treatments that once yielded predictable results now end in deterioration or readmission. The repeated experience of therapeutic failure, often without clear diagnostic guidance or institutional support, fosters professional dissatisfaction, helplessness, and moral injury. For many frontline clinicians, this chronic pressure translates into burnout, a state exacerbated by high patient loads, insufficient infrastructure, and unrealistic expectations from patients and their families. Moreover, the financial and ethical implications of having to prescribe scarce or costly alternatives, such as carbapenems or linezolid, when patients cannot afford them, add another layer of moral conflict. Providers often find themselves negotiating not only clinical outcomes but also economic realities, deciding which patients can access life-saving treatments and which cannot. In this environment, the joy and pride traditionally associated with medical practice begin to fade, replaced by disillusionment and fatigue.

To support healthcare workers in this uphill battle, systemic reforms must prioritize antimicrobial stewardship, strengthen supply chains for essential medicines, and improve access to diagnostic services. Equally important is investing in the mental well-being and continuous education of providers, ensuring they are not only equipped to face resistance-related challenges but also empowered to preserve the integrity of their clinical practice.

## Conclusions

The poorly regulated use of antibiotics in Nigeria represents a critical public health emergency that threatens both individual patient outcomes and the integrity of the national healthcare system. This review has highlighted how a confluence of weak regulatory enforcement, widespread misinformation, economic hardship, and systemic healthcare gaps has created a dangerous environment where antibiotics are routinely misused. The consequences of rising AMR, increased treatment failures, diagnostic uncertainty, and erosion of trust in medical care are no longer theoretical projections but daily clinical realities for healthcare providers across the country.

Unless Nigeria implements coordinated, multisectoral reforms, including strict regulation of antibiotic sales, widespread public education, expanded healthcare access, and robust surveillance systems, the nation risks losing one of the most foundational tools in modern medicine. The future of safe surgical procedures, infection control, maternal health, and routine clinical care depends on urgent action. Policymakers, healthcare professionals, regulatory agencies, and the public must come together to preserve the effectiveness of antibiotics. Failure to act now may result in a grim future where once-treatable infections become untreatable, and basic medical interventions carry unacceptably high risks. Nigeria stands at a critical crossroads in the fight against AMR, and its response will shape the health of generations to come.
